# More intelligent extraverts are more likely to deceive

**DOI:** 10.1371/journal.pone.0176591

**Published:** 2017-04-27

**Authors:** Justyna Sarzyńska, Marcel Falkiewicz, Monika Riegel, Justyna Babula, Daniel S. Margulies, Edward Nęcka, Anna Grabowska, Iwona Szatkowska

**Affiliations:** 1 Institute of Psychology, Polish Academy of Sciences, Warsaw, Poland; 2 University of Social Sciences and Humanities, Warsaw, Poland; 3 Neuroanatomy & Connectivity Research Group, Max Planck Institute for Human Cognitive and Brain Sciences, Leipzig, Germany; 4 Nencki Institute of Experimental Biology, Department of Neurophysiology, Polish Academy of Sciences, Warsaw, Poland; 5 Nencki Institute of Experimental Biology, Laboratory of Brain Imaging, Neurobiology Center, Polish Academy of Sciences, Warsaw, Poland; 6 Institute of Psychology, Department of Philosophy, Jagiellonian University, Kraków, Poland; University of Bologna, ITALY

## Abstract

The tendency to lie is a part of personality. But are personality traits the only factors that make some people lie more often than others? We propose that cognitive abilities have equal importance. People with higher cognitive abilities are better, and thus more effective liars. This might reinforce using lies to solve problems. Yet, there is no empirical research that shows this relationship in healthy adults. Here we present three studies in which the participants had free choice about their honesty. We related differences in cognitive abilities and personality to the odds of lying. Results show that personality and intelligence are both important. People low on agreeableness and intelligent extraverts are most likely to lie. This suggests that intelligence might mediate the relationship between personality traits and lying frequency. While personality traits set general behavioral tendencies, intelligence and environment set boundaries.

## Introduction

In everyday social life, frequent lying, manipulation and taking advantage of others is considered maladaptive and generally condemned by society—big lies may lead to substantial legal, social and personal damages. But not all lies share these adverse characteristics. The most common white lies cause little harm or might even be beneficial for others. Although both types are present in everyday social life, recent research has shown that it is honesty that determines most people’s actions and decisions [1,2] with relatively few ‘prolific liars’ who are responsible for the majority of lies [3]. What are the factors that affect how often people lie and manipulate others?

The tendency to lie is embedded in our personalities—each person has an attitude towards lying in specific situations. Several personality traits (for example, psychopathy) are partially defined in terms of deceiving and manipulating others. Research suggests that different combinations of personality traits increase the likelihood of lying [4–10]. People high in antisocial personality traits—Machiavellianism, narcissism and psychopathy, known collectively as 'the Dark Triad' [11]—lie more often; psychopathy is linked to lies for no reason, narcissism to self-gain lies and Machiavellianism to white as well as self-gain lies [4–6]. Personality traits without antisocial characteristics are also relevant. People high in psychoticism lie more often both to themselves and others [10]. Neurotic people use more self-deception and have a more deceptive communication style [9]. People high on social adroitness and extraversion reported telling more lies during social interactions [5]. In one study the authors demonstrated that people high on manipulativeness and moral disengagement are less likely to refrain from lying when their reputation is at risk [8]. These results confirm that personality traits influence the likelihood of lying in different contexts. But are personality traits the only dispositional characteristics that influence how likely people are to lie?

We propose that general intelligence (*G*) and executive functions [12] might be just as important. Mechanistic models of deception—such as the Working Memory Model of Deception [13], Activation-Decision-Construction Model (ADCM, [14]), Activation-Decision-Construction-Action Theory (ADCAT, [15]) and cognitive neuroscience models of deception [16]—include executive functions as essential components. According to these models, working memory construes false answers, attentional shifts engage when false and true answers are given one after another, and inhibition prevents giving a true answer when the individual wants to lie. Brain imaging studies of deception show a consistent pattern of increased brain activity in executive function-related networks [17,18]. Lying is more difficult for people with Parkinson's Disease, which primarily affects brain systems important for executive functions [19]. Executive functions had been considered as building blocks for *G* [20,21], but later research showed that only working memory updating and storage capacity are relevant [22–24] for both fluid (*Gf*) and crystallized (*Gc*) intelligence.

It seems that both types of *G* are important for the quality and thus effectiveness of lies. The link between *G* and deception frequency was reported in the developmental literature—*G* of 3-year old girls correlated positively with the frequency of their engagement in deception, as reported by the mothers [25]. People with high *G* are better at faking personality questionnaire responses to fit the profile desired by a potential employer than people with low *G* [26]. Vrij, Granhag and Mann [27] highlight three features that might be associated with cognitive abilities and make the individual a better liar: verbal fluency, originality and speed of processing. Verbal fluency is associated with intelligence [28], originality with creativity [29], and speed of processing affects many low-level cognitive functions. High levels of these abilities enable individuals to produce more credible lies with less cognitive load, even in new, unexpected situations. More creative individuals might feel more entitled and engage more often in unethical behaviors [30]. A potential role of *G* for lying is emphasized in Information Manipulation Theory 2 (IMT2, [31]), where everyday lies are viewed as quick and easy solutions where honesty could be problematic. In IMT2, the estimated cognitive load of each potential solution is considered when choosing how to respond to a given situation. This implies that people with higher cognitive abilities should be more likely to choose an option associated with greater cognitive load—they will recognize lying as an option they can easily put into effect. Therefore, the proposed link between cognitive abilities and lying frequency is indirect: deceptive behaviors are reinforced through their effectiveness. Furthermore, people with higher cognitive abilities might be able to make more accurate judgments about the benefits of deceptive behaviors given the context. In adolescents, reports suggest positive relationship between verbal and creative intelligence and arrogant/deceptive interpersonal style [32]. The authors argue that intelligence facilitates the expression of some aspects of psychopathy; abilities such as reading others, manipulating and being able to charm others are unlikely to be executed efficiently without high cognitive abilities. A similar relationship with verbal intelligence was also reported in a clinical sample of adults [33]. These studies have related intelligence to personality traits that are, by definition, linked to deception, but did not assess lying frequency directly. To our knowledge, the link between cognitive abilities and an empirically assessed frequency of lying has never been demonstrated in healthy adults. Here we attempt to fill this gap.

We hypothesized that participants with higher cognitive abilities would choose deceptive behaviors more often than participants with lower cognitive abilities. To test the hypothesis, we designed two new laboratory-based paradigms in which the participant decided whether and when to lie or tell the truth. We chose laboratory-based contexts, so that all participants could be consistently probed. In the first paradigm (used in Experiments 1 and 3), it was explicitly suggested that both honest and deceptive behaviors can lead to achieving the goal of the scenario. In the second paradigm (used in Experiment 2), an incentive to lie was introduced. We also measured individual differences in fluid intelligence, selected executive functions (working memory updating, attention switching and response inhibition) and personality traits [34].

## Methods

In this section, we present the methodology used for each experiment, followed by the statistical framework used for analyses. The tasks in each experiment were performed inside a MRI scanner. The projects presented here addressed two main questions: who is most likely to lie and how do the choices affect the neural correlates of deception and truth-telling. We felt that for clarity, these two aspects of the experiments should be presented separately. Here we present the results pertinent to the question about individual differences.

### Experiment 1

#### Participants

Seventy-six individuals (38 females) participated in the study. The participants’ mean age was 25.36 (SD = 5.01). The subjects were recruited through advertisement posted on an Internet forum (Gumtree). All subjects were Caucasian, native Polish speakers. All of them were right handed and had normal or corrected-to-normal (contact lenses) vision. The study was approved by the University of Social Sciences and Humanities ethics committee. They all signed a written consent before participating in the study.

#### Measurement of individual differences

Fluid intelligence was assessed with the Standard Plus version of Raven’s Progressive Matrices (RPM) [35]. We used a paper-and-pencil version of the test. Participants were given unlimited time to complete the test. The raw scores were converted to centiles based on the Polish norms [36] and used for further analyses.

We used the 3-back task to assess working memory updating ability [37,38]. The stimuli used in 3-back task were abstract objects. We instructed the participants to press a response button when they detected a target—the same stimulus as presented three items before—and refrain from responding otherwise. Lures on positions n-1 and n-2 were also present. Based on performance, we estimated discriminability (d' or d-prime) and bias using Signal Detection Theory [39] methods implemented with a hierarchical Bayesian model [40]. d' indicates how well the person discriminates between signals and noise, bias describes the strategy used when responding. Two levels of hierarchy were included in the model—the group-level and individual-level. This procedure substantially improves the power of estimation. Individual-level means of posterior distributions at were used as performance measures.

Response inhibition was assessed with a custom implementation of Stop Signal Task. The Go trials were digits, excluding 0 and 5. After digit presentation, for 25% of the trials a bracket (the stop signal) surrounding the digit appeared on the screen. The computer program controlling the experiment adjusted the delay of bracket (i.e. Stop Signal Delay, SSD)—increased the SSD by a fixed time after successful inhibition and decreased it after false alarms—so that each participant performed at 50% accuracy. We asked the participants to judge whether the digit was odd or even as quickly as possible, but withdraw from pressing a button when the bracket appeared. We used Stop Signal Reaction Time (SSRT) as the primary performance measure. SSRT were estimated using a Bayesian hierarchical model [41]. Like in the signal detection theory model used for d' and bias estimation, the two levels of hierarchy in the SSRT model correspond to group- and individual-level estimates. Means of posterior distributions at individual-level were used as the response inhibition performance measure.

Attention switching was assessed with a computer-based version of the Stroop task [42]. In the task participants are asked to make a binary choice regarding the color of the presented text. Congruent (e.g. ‘blue’ written with blue font) and incongruent (e.g. ‘blue’ written with red font) stimuli were presented. Although the Stroop task involves several cognitive functions, including pre-potent response inhibition and attentional control, we use it here as a measure of attention switching. To achieve that we calculated median reaction times for consecutive congruent trials and for incongruent trials following congruent trials. We interpret the difference between the two as a measure of attention switching effectiveness.

Personality was assessed by the use the paper-and-pencil version of NEO-Five Factor Inventory (NEO-FFI [34,43]). NEO-FFI consists of 60 items and the results are calculated for five subscales: Neuroticism, Extraversion, Agreeableness, Conscientiousness and Openness to Experience. We decided to use this questionnaire instead of direct measurement of antisocial personality traits for three main reasons. First, NEO-FFI provides a broad description of personality beyond antisocial traits, thus providing more exploratory value. Second, NEO-FFI has been adapted and validated in the Polish language [44,45]. Third, we studied samples of healthy participants. Most tools assessing antisocial traits are clinical, and thus not suitable for testing individual differences in healthy individuals.

#### Deception task

We used the free-choice version of Speed-Dating Task (SDT) [46]. SDT is based on a real-life event, in which participants engage in short conversations. After each conversation, they decide if they want to meet their speed-date for a real date. After completion of such an event, the organizer shares phone numbers to the matched pairs. We have used this concept to introduce social context within the SDT.

**Fig 1:**
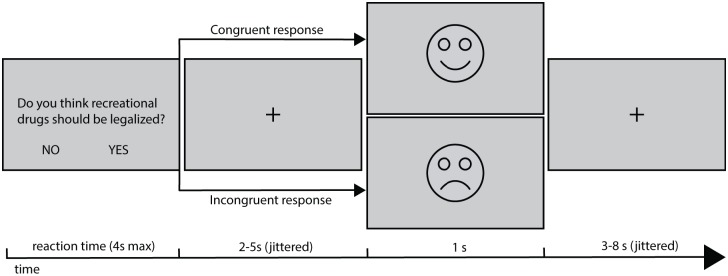
Time course of a single trial in Speed-Dating Task. The received feedback was dependent on consistency of the participant's response with their date's attitudes.

In SDT, participants respond with a yes/no answer to sets of questions asked by different dates that are presented on a screen. The dates are virtual characters made up by the experimenters. Each date ‘asks’ 20 questions about the participant’s attitude towards one of four topics (religion, Weltanschauung, personality or external appearance). Questions related to one topic are asked by two dates, so the participant engages in 8 speed-dates during SDT. For each topic, the two dates asking questions represent an opposite, stereotypical attitude, e.g. one of the dates who ask questions about religion is a very devout catholic, whereas the other is an atheist strongly opposing the church. After each response given by the participant, feedback is displayed on the screen as a frownie or smiley. A smiley indicates that the participant’s response is consistent with their current date’s attitude, while the frownie indicates inconsistency. Each date has a fixed set of response-dependent feedback messages that are contingent with their attitude towards the discussed topic. For a pair of dates with opposite attitudes related to the same topic, the response-feedback mapping is exactly opposite, i.e. if the participant responds the same way for both dates, he/she will receive a smiley on one occasion and a frownie on the other. Looking at feedbacks, participants learn very quickly (after 1–2 questions) what kind of attitude the date represents. An example trial for the Speed-Dating Task is presented in Fig 1.

The goal was to respond to questions in a way that would convince all speed-dates to opt for a real date. It was explicitly stated that this goal could be achieved if the participant remains honest all the time—hoping that the dates will appreciate it—as well as adapt the responses when necessary to appear similar to each date. Thus, the participants had a free choice about the way they wanted to achieve the goal. We refer to the chosen behavior in SDT as ‘strategy’.

We told the participants that they would be paid 50 PLN (approx. 12 EUR) each for the participation in the experiment, but can make up to twice as much if they manage to convince all speed-dates to meet (in fact everyone received 100 PLN for participation).

#### Procedure

The day before participating in the study, all participants filled out an online questionnaire related to their attitudes towards the topics discussed during the dates. At that point, the participants were not informed what the purpose of filling out the questionnaire was, but were explicitly asked to respond honestly. The questionnaire consisted of the same items as in SDT, which had the form of a statement, rather than a question. For each statement, the participant could respond ‘agree’, ‘disagree’ or ‘hard to tell’. The responses given in a questionnaire were used to qualify later responses in SDT as honest or deceptive. Questions for which the participants responded ‘hard to tell’ were excluded from further analyses, although they were presented during SDT.

SDT was performed in a 3T Siemens Trio MRI scanner. The stimuli were displayed on a 27” MRI-compatible LCD monitor placed behind the scanner. The monitor was seen by the subjects through a system of mirrors mounted on the head coil. Stimulus delivery, as well as response recording was controlled by Neurobehavioral Systems Presentation. The participants responded with NeuroNordicLab ResponseGrip response pads held in both hands. Thumbs were used for yes/no responses.

After completion of the MRI protocol, the participants filled out the NEO-FFI personality questionnaire. They were debriefed afterwards and an appointment was made for behavioral testing on another day.

During behavioral testing, the researcher administered the tasks in the following order: 3-back, Stop-Signal Task, Stroop task, Raven’s Test. After the tests were completed the participants received compensation for participation in the experiment.

#### Behavioral strategy calculation

Following the experiment, we classified the responses recorded during SDT into 7 categories. The categories were based on the responses given by respective participants in the pre-study attitude questionnaire and their context within the task:

- Honest consistent (HC) responses—truthful responses consistent with interlocutor's attitude (positive feedback)
- Honest inconsistent (HI)—truthful, but inconsistent with interlocutor's attitude (negative feedback)
- Deceptive consistent (DC)—dishonest responses, but in line with interlocutor's attitude (positive feedback)
- Deceptive inconsistent (DI)—dishonest responses, inconsistent with interlocutor's attitude (negative feedback)
- Adaptive response (AD)—consistent with interlocutor's attitude, without specific attitude of the participant (positive feedback)
- Defiant (DEF)—inconsistent with interlocutor's attitude, without specification of participant's attitude (negative feedback)
- Miss—questions without responses (negative feedback)

We have used only HI and DC responses for strategy calculation:

$$S=\frac{N_{DC}}{N_{DC}+N_{HI}}$$

where *S* denotes strategy for one participant. In other words, strategy is expressed as a fraction of deceptive responses in the context of discordance between the participant’s and date's attitude towards a specific issue. Therefore, strategy reflects how likely the person is to lie, given the opportunity. AD and DEF responses cannot be classified as truths or lies, therefore they are omitted from calculation. DI responses were rare (S1 Fig) and did not provide additional information about the participant’s behavior.

### Experiment 2

#### Participants

44 subjects (22 females) participated in Experiment 2. The mean age of the participants was 22.07 (SD = 2.85). The recruitment process started with e-mails sent to the participants of former fMRI studies in the Nencki Institute. Those who agreed to take part in the experiment were asked to become a team captain, invite some friends to take part in the study as well, and form a team of 6 participants. The aim of this recruitment strategy was to encourage cooperation within teams and competition between teams. To make sure that all the team members met the requirements for MRI scanning, they were asked to fill in an application form posted on a website. Through the website, all the team members were also presented with an advertisement and regulations of the location-based game being part of the study. Once the teams were formed and accepted, all the members were asked to fill in another form and give some personal details that were in fact true answers to autobiographical questions used in the game. All the participants were native Polish speakers, right-handed, with no history of neurological diseases and normal or corrected-to-normal (contact lenses) vision. The study was approved by the University of Social Sciences and Humanities ethics committee. All of them signed a written consent form before participating in the study.

#### Measurement of individual differences

We used the same individual differences measures as in Experiment 1.

#### Deception task & procedure

The context for deception in Study 2 was set in a location-based game designed by MR. In this type of real-world games participants complete tasks with varying difficulty. These tasks usually involve finding items or persons. The tasks were chained, so that the completion of one task led to another task in another physical location.

The location-based game 'Meeting the spy' was organized in Warsaw, in a big city park nearby the Nencki Institute of Experimental Biology. Only one team of 6–8 persons took part in the game each time, so it was organized seven times (additional three times in a pilot study). The game started at the Nencki Institute with the presentation of detailed instructions for the participants. Each participant was given a map and written instructions, and the whole team received a tablet with a special GPS application based on the Geocaching system. The whole team was asked to search for 2 characters, 2 caches and to complete 3 tasks. Creativity and accuracy were assessed, regardless of how long it took them to complete the gameplay. However, the participants were also informed that the score obtained by each team would only partly influence their chances for the final individual financial reward. The main competition would require answering questions about the details of the game individually, during an fMRI experiment. In this way, all the members of the team were encouraged to get properly involved in the game and remember as much as possible. There were two characters engaged in the game and waiting in the park for the teams to describe their tasks and to answer any possible questions.

When all tasks were completed the teams were asked to come back to the Nencki Institute where they were introduced to the process of MR data acquisition and presented with all safety regulations. Also, each participant completed an MRI safety screening questionnaire and the scanning sessions were scheduled for the same day. Each session was preceded by a conversation with an experimenter, IS. In a separate room, participants were given a list of questions about the details of the game and asked to answer truthfully. Then, they were given the instructions (S1 Text) of an upcoming interrogation. Being fully truthful was treated as an evidence of cooperation with the interrogator and guaranteed a low financial reward (approximately 3EUR). Concealing the details of the game guaranteed receiving a high financial reward (approximately 60EUR). However, the interrogator had already received two sources of information: the forms that they completed online and the lists of questions concerning the game that they had just completed. They were instructed to give true answers to these questions to make the interrogator trust them. Subsequently, they were given another list of questions which had not been given to the interrogator. The experimenter discussed all the unclear questions with the participants and pointed out that the questions received by the interrogator formed several topic categories. This way, the participants could easily remember when they were supposed to tell the truth. Finally, the participants were left alone for 10 minutes to compare two groups of questions and choose the preferable strategy.

During a functional scanning sequence, the participants saw the same instructions on the screen. They were asked 120 questions which they had already known. Some of these questions were autobiographical (based on the online forms), others addressed their witness status (eg. Have you seen …?), or their participant experience (e.g. Have you taken part …?). Questions were displayed until the answer yes/ no was given by pressing the button, but no longer than 6s. The questions were separated by an inter-stimulus interval of 1–12.5s.

#### Behavioral strategy calculation

There were several criteria for classifying the questions in Experiment 2. The first criterion was related to whether the question addressed the events during the location-based game in a witness or participant role. The third option here was autobiographical questions for which the participants were supposed to respond honestly; this option was not considered for calculating the strategy. The second criterion was the veracity of the response itself. The third criterion was whether the response was given in concordance with the instructions (i.e. don't lie when you declared the facts beforehand). This led to the following classification:

- Participant, honest adequately (PHA)
- Participant, honest inadequately (PHI)
- Participant, deceptive adequately (PDA)
- Participant, deceptive inadequately (PDI)
- Witness, honest adequately (WHA)
- Witness, honest inadequately (WHI)
- Witness, deceptive adequately (WDA)
- Witness, deceptive inadequately (WDI)
- Misses—questions without response

We used the following equation to estimate strategy:

$$S=\frac{N_{PDA}+N_{WDA}}{N_{PDA}+N_{WDA}+N_{PDI}+N_{WDI}}$$

where S denotes strategy for one participant. In other words, strategy is expressed here a fraction of deceptive responses for questions not revealed to the interrogator in the questionnaire.

### Experiment 3

#### Participants

34 subjects (12 females) participated in Experiment 3. The mean age was 23.3 (SD = 2.62) The participants were recruited by a social media group related to cognitive neuroscience. They all signed a written consent form before participating in the study. The group consisted mainly of undergraduate students. The study was approved by the University of Social Sciences and Humanities ethics committee.

#### Measurement of individual differences

In Experiment 3, the set of psychological measures of cognitive abilities was slightly altered. The same constructs were investigated. For fluid intelligence, we used Raven’s Advanced Progressive Matrices (RAPM). The change was motivated by a plan to recruit a rather homogeneous sample of university students. For attention switching, we used a custom implementation of continuous counting task [47]. In this task participants were required to count different objects presented alone (big or small squares) and keep a running count of each type of object. After a variable number of objects had been presented, participants were asked to report their counts for each object separately. We calculated the accuracy of the counts. Measures of working memory performance (3-back), response inhibition and personality remained the same as in previous experiments.

#### Deception task & procedure

We used the free-choice SDT as in Experiment 1, but introduced slight modifications. First, during the SDT questions to which the participants did not have an opinion (i.e. answered ‘don’t know’ in the attitude questionnaire) did not appear during the task. Second, the participants received a fixed gratification of 50 PLN (≈12 EUR) and the instructions did not state any additional rewards depending on the number of convinced dates. The procedure was similar to Experiment 1.

#### Strategy estimation

The responses were classified according to the same scheme as in Experiment 1. AD and DEF response types are present here, because the questions for which the participants did not declare a clear attitude in the questionnaire were removed from the main experiment. We calculated the strategy in the same way as in Experiment 1.

#### Data analysis

We conducted an integrated analysis of the results of the three studies with a full Bayesian inference framework. Bayesian framework allows for formal incorporation of prior knowledge into the data analysis process, making it ideal for integrating results of several studies. In other words, the Bayesian framework allows the researchers to integrate knowledge about results from the previous experiments (priors) with the current data (likelihood) to produce a consensus of the two (posterior). The posterior knowledge from one study can then be used as a prior for another.

In Experiment 1, for each parameter the prior is a Gaussian distribution with μ = 0 and σ = 1. This prior can be considered as informative and causes shrinkage of uncertain parameter estimates towards zero. The motivation for using this prior is the assumption that very high effect sizes are unlikely given the noisy nature of psychological measurements conducted here. The posterior distributions of parameter estimates were updated with the data from Experiment 2 and Experiment 3. Weakly informative prior was used for the intercept in each experiment (a Gaussian with μ = 0 and σ = 1), because the base probability of choosing a deceptive behavior varied between experiments. The posterior distributions after all updates were used as the basis for inference.

We used a linear logistic regression model for statistical inference. Each variable was normalized (z-scored) before entering the model. Although the dependent variables used in all three studies could be expressed as 'continuous' in the range 0–1, their bimodal distribution indicated that binarizing into two discrete categories (honest/deceptive) would allow us to produce a more accurate statistical model. Thus, for each experiment, the estimated strategy was binarized with the cut-off point at 0.5–0 indicated complete honesty and 1 complete deception. For each parameter, we report both the mean, as well as 95% credible interval (95% CI) of the posterior parameter estimate distribution. We do not report Bayes Factors because of their high dependency on prior specification. The posteriors reported here can be updated when more data is acquired.

For statistical modeling, we used R version 3.3.0 [48] with RStanARM [49] version 2.12.1 high-level interface for Stan [50] package. All analysis scripts, as well as anonymized raw data are available on https://github.com/mfalkiewicz/cognition_personality_deception. The results of the analyses are fully reproducible.

#### Missing and removed data

The combined number of participants in all the three studies was 154. However, complete data was available only for 102 subjects, which were included in the analyses reported below. The primary reason for this is the fact that analytical methods used here required complete data to include the participant in the analysis. Missing data were randomly distributed across participants, therefore the amount of usable data decreased dramatically.

For 6 subjects, the data about their behavior during the deception task was not available due to technical problems with response pads—the responses were not recorded. RPM scores were not available for 3 subjects. The data related to 3-back task performance was not available for 18 subjects, of whom 13 participated in Experiment 1. The data from the Stop Signal Task was not available for 26 participants, of whom 20 participated in Experiment 1. This large amount of missing data was predominantly due to either technical problems with the equipment (response pads) or software. Lastly, NEO scores were unavailable for 11 participants, all participating in Experiment 3. This was because NEO scores were assessed sometime after the completion of the experiment and not all participants could be reached.

We removed 6 subjects from the analyses in Experiment 2, because they did not comply with the instructions, i.e. deceived in every question.

We have decided to present the results of such a highly-reduced sample to consider all measured variables. However, to verify the robustness of the results, we performed the same data analysis, but we took only fluid intelligence and personality scores into consideration. This analysis, which includes 135 participants gives convergent results with the analyses presented here, showing even stronger effects. The results of this analysis are presented and discussed in S1 Table.

## Results and discussion

### Strategies

**Fig 2:**
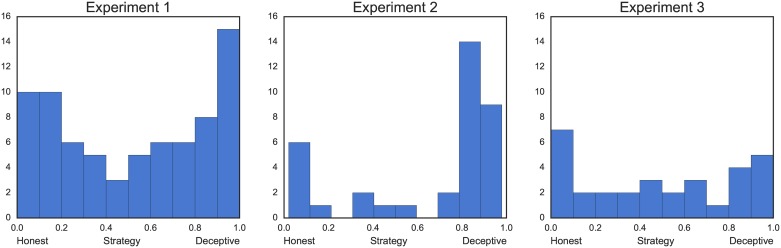
Histograms representing the distribution of strategies chosen by participants in each experiment. For analysis, the strategies were binarized with threshold = 0.5.

In all three experiments, we observed a very similar bimodal distribution of strategies (Fig 2). In E1 and E3 most participants chose to either remain honest almost all time, or be deceptive, with relatively few intermediate strategies. In E2, majority of the participants chose to attempt a deceptive strategy. This is probably because lying in E2 could lead to substantial monetary gain, while nothing could be gained from honesty. Despite a clear advantage of the deceptive strategy, few participants still chose to remain honest most of the time. Distributions of each response within each experiment are presented in S1 Fig.

### Individual differences and deception odds

The process of updating beliefs about parameter estimates is illustrated in S2 Fig. Markov Chains converged for all parameters (Rhat = 1). The parameter estimates for the model reported here are presented in S1 Table.

### Demographic measures

Age and biological sex did not show any significant relationship with deception odds. Although the posterior distribution of age has the highest density at 0.38, it also has very wide credible intervals (95% CI: [-0.54–1.3]). Therefore, we cannot conclude any significant role of biological sex for deception odds. Age has a positive relationship with deception odds—elder people are more likely to choose a deceptive strategy, but the effect is relatively small (M = 0.15, 95% CI: [0.01–0.29]).

### Fluid intelligence and extraversion

**Fig 3:**
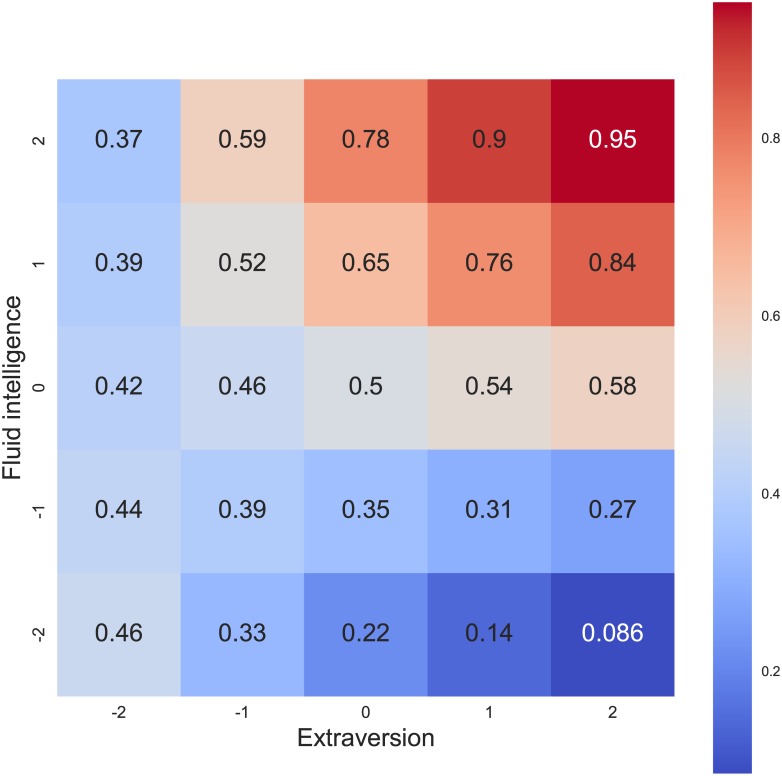
Gf x extraversion interaction. The numbers in cells denote the probability of choosing a deceptive strategy, with assumption that a person with average IQ and extraversion has the probability 50%. The values in axes represent standard deviations w.r.t. the mean value.

The Raven's Progressive Matrices score has significant relationship with deception odds. One standard deviation increase in RPM results in an increase of log-odds of choosing deceptive strategy by 0.63 (95% CI: [0.49–0.77]). To give a more intuitive understanding of these numbers, we can convert them to probabilities. For all subsequent conversions, we will assume that a person with an average RPM score has a 50% probability of choosing a deceptive strategy. Increase in log odds by 0.63 means that a person with an RPM score 1 standard deviation above the mean will have the probability of choosing a deceptive strategy equal to 65% and a person with 2 SD above the mean: 78%. We also found an interaction of RPM score with extraversion (M = 0.36, 95% CI: [0.24–0.49]). However, extraversion alone has a relatively weak relationship with deception odds (M = 0.17, 95% CI: [0.03–0.31]). A graphical representation of this relationship is presented in Fig 3. For introverts, changes in intelligence have little impact on deception odds. On the other hand, the dependency between IQ and deception odds is strong for extraverts. Highly intelligent extraverts are the most likely to choose a deceptive strategy and the least intelligent extraverts are least likely.

The previously reported relationship between extraversion and frequency of lying are contradictory. A significant relationship was found in one study [5] and no significant relationship was found in another [10]. Functions that lies serve might be especially important for extraverts because of their high level of sociability [5]. The novelty of the work presented here is the finding that the effect of extraversion on lying frequency depends on intelligence (or, conversely, effect of fluid intelligence on lying frequency depends on extraversion level). This finding links the well-established relationship between personality traits and deception frequency with cognitive aspects of lying. The role of fluid intelligence here is unclear. It might have an indirect effect on lying frequency by making the person a more successful liar, which in turn reinforces the use of deceptive behaviors [31]. Alternatively, one might speculate that in the context of current studies that higher fluid intelligence made the participants pursue deception, realizing it to be the most successful strategy.

The mechanism responsible for the presence of such interaction also remains speculative. Given that high cognitive abilities are important for a good liar [27] and that extraverts are more sensitive to positive outcomes of social lies [5], high levels of both might make a person especially inclined to deceive. Introverts would be less sensitive to potential social rewards, therefore less prone to deception. Less intelligent people would be less likely to deceive because they do not have a successful history of lying and/or are less likely to perceive deception as beneficial. Less intelligent extraverts might have had an unsuccessful history of deceptions in social context, therefore might be less likely to pursue such behaviors.

### Other personality traits

Agreeableness has a significant, negative relationship with deception odds (M = -0.65, 95% CI: [-0.79 –-0.5]). The more agreeable the person is, the less likely he/she is to choose a deceptive strategy. A person with agreeableness 1 SD above the mean has the probability 34% of choosing a deceptive strategy, whereas for a person with agreeableness 2 SD below the mean the probability drops to 21%. Agreeableness does not interact with RPM (M = 0.08, 95% CI: [-0.07–0.22]). Neuroticism (M = -0.21, 95% CI: [-0.35 –-0.06]) and conscientiousness (M = -0.33, 95% CI: [-0.47 –-0.19]) are both negatively related to deception odds. Openness to experience is positively related to deception odds (M = 0.15, 95% CI: [0.01–0.29]), i.e. persons with higher openness are more likely to choose a deceptive strategy. There are no interactions of personality dimensions mentioned above with fluid intelligence.

Significant relationships between all measured personality dimensions and deception odds are pleasing, because they support the face validity of deception contexts investigated here. All Big Five measures are correlated with Dark Triad traits, with agreeableness, conscientiousness and neuroticism showing negative correlations and openness to experience and extraversion showing a positive relationship [11]. Therefore, this exact pattern of relationships is related to higher Machiavellianism, narcissism and psychopathy. Furthermore, the personality dimension most strongly related to deception odds in the presented studies, agreeableness, shows strongest and most consistent negative correlation with all Dark Triad traits [51]. Although Dark Triad traits were not measured here directly, this pattern of results strongly suggests the behaviors chosen during the deception tasks are related to the level of participants' antisocial traits.

### Other cognitive measures

No other cognitive measures showed a convincing relationship with deception odds. SSRT (M = 0.03, 95% CI: [-0.1–0.17]), switching costs in Stroop task (M = 0, 95% CI: [-0.27–0.28]), as well as bias in 3-back task (M = 0.03, 95% CI: [-0.25–0.31]) have a relatively narrow posterior distribution centered around zero. The posterior distribution for accuracy in the continuous counting task is very wide (M = 0.22, 95% CI: [-0.51–0.96]) and cannot be considered as significantly different from zero. Discriminability in the 3-back task has a slightly positive mean (M = 0.1, 95% CI: [-0.03–0.23]) of the posterior distribution, but too large portion of the distribution crosses zero to consider it significant. The process of updating the posterior is presented in S2 Fig.

This result might be explained by the fact that involvement of executive functions depend largely on the type of deception [2,15,31,52]. Probably the executive functions we decided to measure are more important to other types or aspects of deception (i.e. high-stakes lies) and their impact is more clearly visible in the instructed lying paradigms. Alternatively, the tests employed in the reported studies capture only some aspects of constructs they refer to [38]. Hence, it might be the case that other aspects of executive functions are important for different types of deception.

## General discussion

Previous research has clearly indicated that the tendency to engage in deceptive behaviors is related to specific personality traits and contextual factors [3–6,8,10]. Intellectual abilities are important for the quality of lies [26,31] and are related to deceptive communication style [32,33,53]. In the work presented here we investigated whether intellectual abilities are also relevant for the frequency of lying. For this purpose, we performed three laboratory-based experiments in which the subjects were free to choose whether they want to behave deceptively or not. In the context of location-based game, the deceptive strategy was clearly advantageous, because of financial incentive to lie. Within the Speed-Dating Task the value of each strategy was ambiguous and assessed subjectively. Using a Bayesian statistical framework, we combined the results of all three experiments. The results replicate most of the previously reported relationships between personality traits and frequency of lying in social interactions. The results also strongly indicate that fluid intelligence has a significant relationship with deception odds and interacts with extraversion. Highly intelligent extraverts are most likely to engage in deceptive behaviors. Highly intelligent introverts as well as extraverts with lower intelligence are least likely to deceive.

We can think of deception as a tool for adapting to environmental demands. In some situations, individual goals can be achieved through honesty, in others they can't. The decisions are based on subjective judgement—people take into account the expected value of available behavioral options when deciding whether to lie or not [15]. According to IMT2 [31], people also take into account the expected difficulty of lying in a given situation—presumably in the light of their general skill in lying and knowledge about the other person. Therefore, IMT2 treats G as a mediator for situational factors and personality traits. The data collected here does not allow to make causal claims, but the mediator interpretation is most likely.

In the Speed-Dating Task used in Experiments 1 and 3, adapting to the date's perspective (i.e. lying) led to positive feedback. Although the instruction clearly stated that positive feedback merely indicates consistency with the date, the smiley associated with such responses could be interpreted as a form of external reward. This might explain why intelligent extraverts were most likely to pursue deceptive behavior—they wanted the reward and subjectively judged that they could be effective in getting it.

The results suggest a general form of interaction between personality and intelligence for decision making about lying and truth-telling. While personality traits set general behavioral tendencies, intelligence and environment set boundaries. People who look for external rewards (extraverts) will use deception only if they believe they will be successful.

We must remember that intelligence is not the main factor which affects the decision to lie or not. Our research shows that high intelligence combined with other personality traits increases the likelihood of lying. Therefore, a model explaining the dispositional factors that affect decisions to lie should include intelligence as one of its components.

### Study limitations

Although this study provides new insights into the role of cognitive abilities for choosing insincere behaviors, there are several limitations that need to be considered. First, we have probed participants’ behavior in contexts consistent within each study, which removes a large portion of variability due to the changing context of everyday life. One might ask to what degree these laboratory-based settings reflect the behaviors in individual’s daily lives. We did not try to convince the participants that the dates are real; depending on the personality and intelligence, participants might had interpreted the settings as real or artificial, which could affect decisions about their behavior. The situation was highly artificial, although surprisingly many participants reported strong emotions during the study. Furthermore, only one form of deception was available (behaviors were expressed through button presses). Despite that, a highly significant relationship of behavior in such conditions with personality traits—expected to be related to real-life deception frequency—alleviates the concerns about face validity. However, alternative methods of assessing the frequency of lying should be employed and compared with results obtained here. Second, the results are based on a relatively small sample size in Experiments 2 and 3, which lead to their relatively small impact on final results. Third, the sampling of deception contexts was not uniform—70 of 102 participants took part in the Speed-Dating Task. Therefore, the context of Speed-Dating Task has more impact on the combined results. Both sample sizes as well as sampling of deception context should be more balanced in future studies using similar methodology. Fourth, we included mainly young university students in this study. Despite these limitations, we believe that these results are valid and provide a unique contribution to research on psychological factors related to decisions to deceive others.

## Supporting information

S1 Text Task instructions. The original instructions were presented in Polish.
(PDF)

S1 Fig The counts of each response type for each experiment. The whiskers indicate minimum/maximum, the bottom/up of each box indicates 1st and 3rd quartile, respectively and the horizontal line inside the box indicates the median.
(PDF)

S2 Fig The evolution of posterior distributions after each experiment. Posteriors from previous experiments were used as priors in the following experiments.
(PDF)

S1 Table The parameters of a full and reduced model. The full model includes all measured variables, but due to missing values includes fewer samples (reported in the main body of the manuscript). The reduced model excludes the variables with many missing values, but includes more samples.
(PDF)
