# Supplementary material for: More intelligent extraverts are more likely to deceive
Source: PLoS One. 2017 Apr 27;12(4):e0176591. doi: 10.1371/journal.pone.0176591 (PMC5407751; doi:10.1371/journal.pone.0176591)
Supplement: S1 Text — The original instructions were presented in Polish. (PDF) [file pone.0176591.s001.pdf]

S1 Text. Task instructions. The original instructions were presented in Polish.

Speed-Dating Task (Experiments 1 & 3)

You are taking part in a study about social interactions. During the task, you will participate in so-called 'speed-dates' with different persons. The goal of speed-dating is to convince the other person to have a real date with you. Your goal is to convince every one of your dates to have a real date with you. (After completion of the study you can receive a financial reward. The amount that you will receive will depend on the number of dates that will want to meet with you) *.
Each of your interlocutors will ask questions related to one of few topics. You will respond either YES (right hand) or NO (left hand). You will see a hint on the screen which will indicate which side corresponds to which response.
Soon after you respond, you will receive feedback indicating whether your response was consistent or inconsistent with the attitude or preference of the current date. A smiley indicates consistency, a frownie – inconsistency. You don't have to adjust your responses to each date, because he/she might find a person with opposite attitudes interesting and still want to have a real date. Nevertheless, you can also adjust your responses to match the dates attitudes preferences, because this as well might convince the date to meet with you. Try to respond as soon as possible – the time to respond is limited.
Remember, right hand – YES response, left button – NO response.

* - The sentence in the brackets was omitted in E3.

Location-based Game (Experiment 2)

In a moment, you will be interrogated.

You can convince the interrogator that you are innocent if you succeed to conceal more details of the location- based game “Meeting the spy” than the other members of your team. If you persuade the interrogator that the game had a different course than it really had, he will acknowledge that you are not a spy and you will receive a high financial reward. However, the interrogator has already had a certain knowledge about you and about the course of the location-based game “Meeting the spy”, as he had received the form that you completed on-line and the questionnaire concerning the game that you have just completed. Should you give true answers to the questions included in the form and the questionnaire, the interrogator would believe all your answers.

You can also cooperate with the interrogator and admit everything. If you cooperate and inform him about all the details of the game, you would receive a small reward (10% of the award for the person acknowledged innocent).

You will answer the questions concerning the caches, people and tasks that you performed. The coherence of the given answers (the answers should not be contradictory) and the time of answering (the sooner the better) will be assessed. You will give your answers by clicking the buttons YES or NO.

Only one member of your team can be acknowledged innocent and only one can be acknowledged cooperative.

The interrogation will start in a minute.
