# Supplementary material for: More intelligent extraverts are more likely to deceive
Source: PLoS One. 2017 Apr 27;12(4):e0176591. doi: 10.1371/journal.pone.0176591 (PMC5407751; doi:10.1371/journal.pone.0176591)

S1 Fig. The counts of each response type for each experiment. The whiskers indicate minimum/maximum, the bottom/up of each box indicates 1st and 3rd quartile, respectively and the horizontal line inside the box indicates the median.

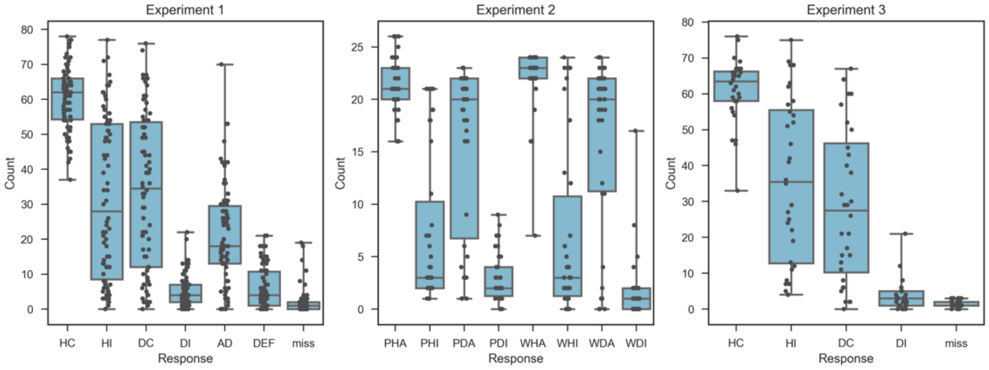

Supplement: S1 Fig — The whiskers indicate minimum/maximum, the bottom/up of each box indicates 1st and 3rd quartile, respectively and the horizontal line inside the box indicates the median. (PDF) [file pone.0176591.s002.pdf]
