# Supplementary material for: More intelligent extraverts are more likely to deceive
Source: PLoS One. 2017 Apr 27;12(4):e0176591. doi: 10.1371/journal.pone.0176591 (PMC5407751; doi:10.1371/journal.pone.0176591)

S2 Fig. The evolution of posterior distributions after each experiment. Posteriors from previous experiments were used as priors in the following experiments.

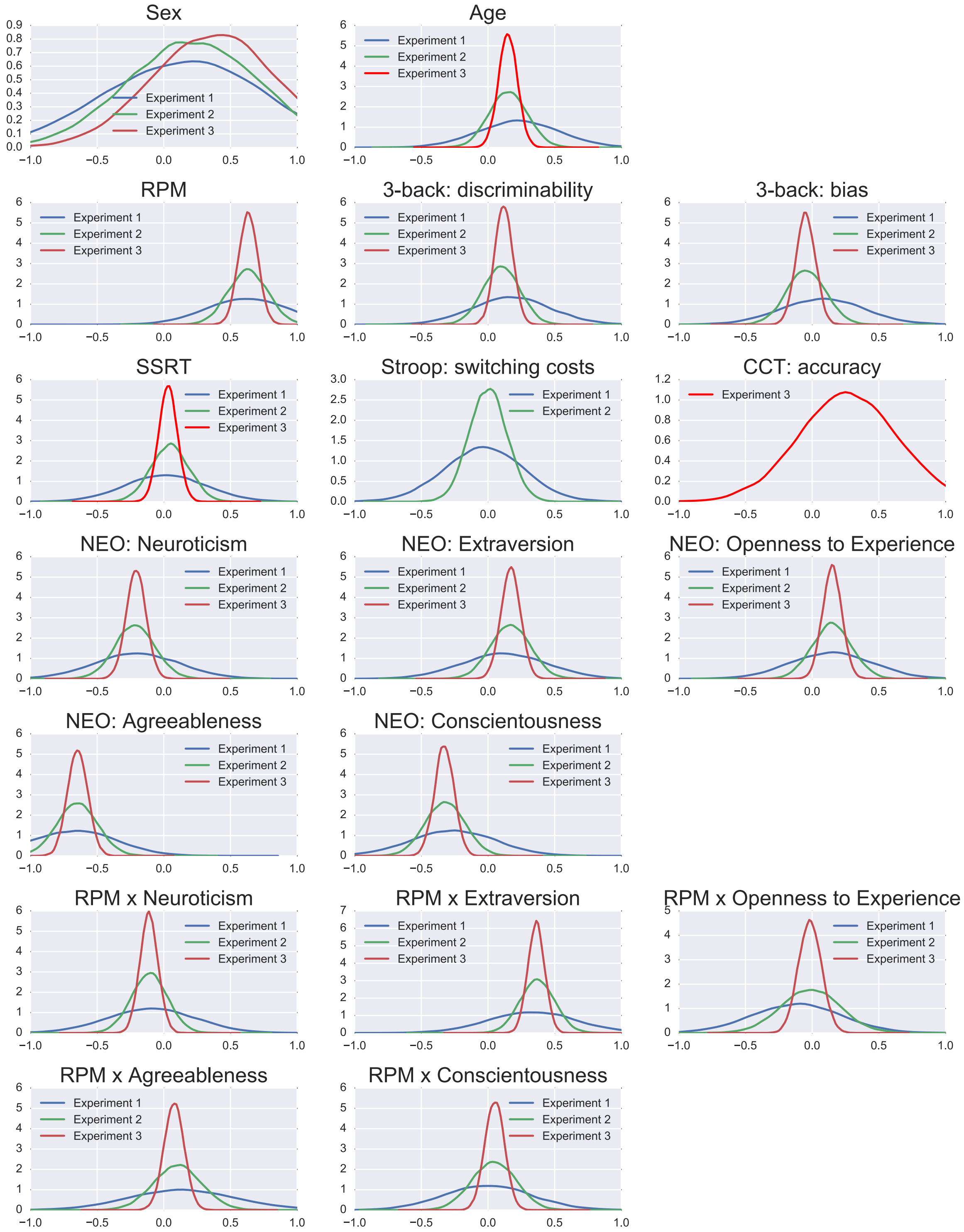

Supplement: S2 Fig — Posteriors from previous experiments were used as priors in the following experiments. (PDF) [file pone.0176591.s003.pdf]
