# Supplementary material for: More intelligent extraverts are more likely to deceive
Source: PLoS One. 2017 Apr 27;12(4):e0176591. doi: 10.1371/journal.pone.0176591 (PMC5407751; doi:10.1371/journal.pone.0176591)
Supplement: S1 Table — The full model includes all measured variables, but due to missing values includes fewer samples (reported in the main body of the manuscript). The reduced model excludes the variables with many missing values, but includes more samples. (PDF) [file pone.0176591.s004.pdf]

S1 Table. The parameters of a full and reduced model. The full model includes all measured variables, but due to missing values includes fewer samples (reported in the main body of the manuscript). The reduced model excludes the variables with many missing values, but includes more samples.

Variable	Full model (N = 102)	Reduced model (N = 135)
Age	0.38 [-0.54 – 1.3]	-0.21 [-0.33 – -0.09]
Sex	0.15 [0.01 – 0.29]	0.2 [-0.6 –0.99]
RPM	0.63 [0.49 – 0.77]	0.69 [0.56 – 0.81]
SSRT	0.03 [-0.1 – 0.17]	
Stroop	0 [-0.27 – 28]	
3-back: d'	0.1 [-0.03 - 0.23]	
CCT	0.22 [-0.51 – 0.96]	
NEO: N	-0.21 [-0.35 – -0.06]	-0.4 [-0.53 – -0.27]
NEO: E	0.17 [0.03 – 0.37]	0.12 [-0.01 – 0.24]
NEO: O	0.15 [0.01 – 0.29]	0.25 [0.13 – 0.37]
NEO: A	-0.65 [-0.79 – -0.5]	-0.64 [-0.77 – -0.51]
NEO: C	-0.33 [-0.47 – -0.19]	-0.3 [-0.42 – -0.18]
RPM x NEO: N	-0.11 [-0.25 – 0.02]	0.1 [-0.06 – 0.25]
RPM x NEO: E	0.36 [0.24 – 0.49]	0.42 [0.3 - 0.54]
RPM x NEO: O	-0.01 [-0.18 – 0.15]	0.07 [-0.07 – 0.22]
RPM x NEO: A	0.08 [-0.07 – 0.22]	-0.18 [-0.29 – -0.08]
RPM x NEO: C	0.05 [-0.09 – 0.19]	0.18 [0.06 – 0.3]

The reduced model is convergent with the results of the analysis with the conservative missing data removal strategy presented in the main body of the manuscript. We will present each effect and briefly discuss its relationship with the model reported in the main text.

For demographic variables, the parameter estimate for sex remained very uncertain and thus also cannot be considered significant. On the other hand, age has shown a significant, negative relationship with deception odds (M = -0.21, 95% CI: [-0.33 – -0.09]. The effect of RPM on deception odds became even stronger than in the main analysis and was 0.69 (95% CI: [0.56 – 0.81]). This confirms that the main finding of the work presented here is robust. The same goes for personality dimensions - all effects had the same signs and very similar parameter estimates.

The interaction effect of RPM with Extraversion was almost identical to the effect reported in the main text (M = 0.42, 95% CI: [0.3 – 0.54]). However, additional interactions which could be considered as significant have emerged: positive interaction with Conscientiousness (M = 0.18, 95% CI: [0.06 – 0.3]) and a negative interaction with Agreeableness (M = -0.18, 95% CI: [-0.29 – -0.08]). Although the effect for Agreeableness

has a consistent sign with the analyses reported in the main text, the sign for Conscientiousness changes from negative to positive. We do not further interpret these effects because of their inconsistency between analyses. However, their presence suggests even more complicated interactions between fluid intelligence and personality for decisions about strategies used in social interactions.
